# Targeting Circulating lncRNA ENST00000538705.1 Relieves Acute Coronary Syndrome via Modulating ALOX15

**DOI:** 10.1155/2022/8208471

**Published:** 2022-05-06

**Authors:** Hao Chen, Shiwei Huang, Fanlu Guan, Sisi Han, Fanhao Ye, Xun Li, Liyi You

**Affiliations:** ^1^Department of Cardiology, The Third Affiliated Hospital of Shanghai University (Wenzhou People's Hospital), Wenzhou, 325000 Zhejiang, China; ^2^Department of Cardiology, The First Affiliated Hospital of Soochow University, Suzhou, 215006 Jiangsu, China

## Abstract

**Objective:**

Acute coronary syndrome (ACS) is the most dangerous and deadly form of coronary heart disease. Herein, we aimed to explore ACS-specific circulating lncRNAs and their regulatory mechanisms.

**Methods:**

This study collected serum samples from ACS patients and healthy controls for microarray analysis. Dysregulated circulating lncRNAs and mRNAs were determined with |log2fold − change| > 1 and *p* < 0.05. lncRNA-mRNA coexpression analysis was carried out. ENST00000538705.1 and ALOX15 expression was further verified in serum specimens. In human coronary artery endothelial cells (HCAECs), ENST00000538705.1 and ALOX15 were knocked out through transfecting specific siRNAs. Thereafter, proliferation and migration were investigated with CCK-8 and wound-healing assays. Myocardial infarction rat models were established and administrated with siRNAs against ENST00000538705.1 or ALOX15. Myocardial damage was investigated with H&E staining, and serum TC, LDL, and HDL levels were measured.

**Results:**

Microarray analysis identified 353 dysregulated circulating lncRNAs and 441 dysregulated circulating mRNAs in ACS. Coexpression analysis indicated the interaction between ENST00000538705.1 and ALOX15. RT-qPCR confirmed the remarkable upregulation of circulating ENST00000538705.1 and ALOX15 in ACS patients. In HCAECs, ENST00000538705.1 knockdown lowered the expression of ALOX15 but ALOX15 did not alter the expression of ENST00000538705.1. Silencing ENST00000538705.1 or ALOX15 weakened the proliferation and migration of HCAECs. Additionally, knockdown of ENST00000538705.1 or ALOX15 relieved myocardial damage, decreased serum TC and LDL levels, and elevated HDL levels in myocardial infarction rats.

**Conclusion:**

Collectively, our findings demonstrate that circulating ENST00000538705.1 facilitates ACS progression through modulating ALOX15, which provide potential targets for ACS treatment.

## 1. Introduction

Acute coronary syndrome (ACS), including acute myocardial infarction (AMI) and unstable angina (UA), is a highly dangerous and fatal deadly form of coronary heart disease, which is mainly triggered by ruptured atherosclerotic plaques and thrombosis [1]. In a large proportion of cases, this syndrome is the cause of acute heart failure [2]. Despite advances in treatment, ACS patients are at high risk of morbidity and mortality [3]. Approximately 20% of patients who successfully received percutaneous coronary intervention had relapsed adverse cardiovascular events within 3 years [4]. Therefore, more effective, safer, and more affordable treatments are needed to reduce the risk of cardiovascular events and death in patients with ACS.

Genomic and transcriptomic analysis suggests that nearly 2% of the human genome is a protein-coding sequence while most of the genome is noncoding RNA (ncRNA) that is classified as small ncRNA and long ncRNA (lncRNA) [5, 6]. lncRNAs with a structure of over 200 nucleotides modulate gene expression and participate in diverse pathophysiological processes [7, 8]. On the basis of the stability of lncRNAs in the blood and other body fluids, they are reliable biomarkers for disease diagnosis [9–11]. Emerging evidence demonstrates that several lncRNAs participate in modulating ACS initiation and progression [12]. For instance, lncRNA ANRIL triggers myocardial cell apoptosis in AMI through IL-33/ST2 signaling [13, 14]. Aging-relevant antiapoptotic lncRNA Sarrah accelerates recovery from AMI [15]. lncRNA ZFAS1 facilitates intracellular Ca^2+^ overload and contractile dysfunction in myocardial infarction (MI) mouse models [16]. Additionally, lncRNAs LNC_000226 and MALAT1 have been determined as diagnostic markers of UA [17]. Despite this, knowledge of circulating lncRNAs and their functions in ACS progression is still in its infancy. In this study, we performed microarray expression profiling to identify differences in circulating lncRNA and mRNA expression patterns between ACS patients and healthy subjects. This study was aimed at determining ACS-specific circulating lncRNAs and analyzing the potential biological functions of lncRNAs during ACS progression.

## 2. Materials and Methods

### 2.1. Patients and Samples

This study was approved by the Ethics Committee of the Third Affiliated Hospital of Shanghai University (Wenzhou People's Hospital) (KY-2017029). All patients signed written informed consent. This research was conducted in accordance with the guidelines of the Declaration of Helsinki. From June 2017 to October 2017, whole blood samples from 9 patients with ACS and 9 healthy participants were collected in the Third Affiliated Hospital of Shanghai University (Wenzhou People's Hospital). The diagnostic criteria of ACS were as follows: patients had stenosis (at least one main coronary artery ≥ 50%) confirmed by coronary angiography and/or met the AMI criteria (typical clinical symptoms, elevated cardiac enzyme levels, and a representative set of electrocardiography (ECG)). The inclusion criteria were as follows: (1) patients met the diagnostic criteria, (2) patients were 35-75 years old, and (3) patients provided informed consent. The exclusion criteria were as follows: (1) patients had comorbid diseases, such as cardiomyopathy, valvular heart disease, severe arrhythmia, heart failure, and other concomitant diseases; (2) patients had data collection difficulties, such as religious or language barriers; and (3) patients were pregnant or breastfeeding. Within 3-5 hours after the onset of symptoms but before angiography, venous blood samples were collected from each subject by anterior elbow venipuncture. The whole blood sample (2 ml) was directly collected into a test tube containing ethylenediaminetetraacetic acid (EDTA), and then, a red blood cell lysis buffer was added and other blood components were removed through centrifugation at 3000 g at 4°C for 5 minutes within 2 hours. Next, 1 ml of TRIzol was added, and the sample was transferred to a RNase/DNase-free tube and stored at -80°C.

### 2.2. Microarray Analysis

Microarray analysis was achieved by Beijing Boao Jingdian Biotechnology Co., Ltd. (China). Briefly, extracted RNA from serum samples of 4 ACS and 4 healthy subjects was reverse transcribed into complementary DNA (cDNA). Additionally, cRNA was synthesized by reverse transcription. Fluorescent labeling, microarray hybridization, microarray cleaning, and scanning were presented. GeneSpring software (version 13.0; Agilent) was adopted for analyzing lncRNA and mRNA microarray data for data aggregation, normalization, and quality control. Differentially expressed lncRNAs and mRNAs were screened in accordance with fold change ≥ 2 and Benjamini-Hochberg-corrected *p* value < 0.05. Moreover, the Adjust Data function of CLUSTER 3.0 software was utilized to perform log2 conversion on the data and determine the median of lncRNAs or mRNAs. Additionally, the data were further analyzed through hierarchical clustering with average linkage. The clustering results were visualized using Java TreeView software [18]. Transcription factors of differentially expressed lncRNAs were predicted with the Match-1.0 Public transcription factor prediction tool. The binding of the 2000 bp and 500 bp regions upstream of the start site of each lncRNA with the transcription factor was predicted. On the basis of the Pearson correlation analysis between differentially expressed lncRNAs and mRNAs, a lncRNA-mRNA coexpression network was constructed in accordance with |correlation coefficient| ≥ 0.99 and *p* < 0.05. Downstream targets of differentially expressed lncRNAs were also determined through cis-lncRNA prediction and trans-lncRNA prediction. The prediction of cis-lncRNA was carried out through close correlation with a set of expressed protein-coding genes utilizing |correlation coefficient| ≥ 0.99 and *p* < 0.05. At the genomic locus where the lncRNA was located, the protein-coding gene and the lncRNA were within 10 kb along the genome. Therefore, “cis” referred to the regulation mechanism of the same locus, including the antisense lncRNA-mediated regulation of protein-coding genes encoded at the same locus. The complete sequence of lncRNA was compared with the 3′UTR of the coexpressed mRNA on the basis of the genome data from the UCSC website (http://hgdownload.cse.ucsc.edu/admin/exe/) [19]. The networks of transcription factor-lncRNA, lncRNA-mRNA, and lncRNA-downstream target were established with Cytoscape software [20]. In the network, degree centrality was defined as the number of links between one node and another node. Degree was the simplest and most important measure of gene centrality in a network of relative importance.

### 2.3. Real-Time Quantitative PCR (RT-qPCR)

RNA extraction was conducted with the MiniBEST Universal RNA Extraction Kit (Takara, China). Total RNA was reverse transcribed into cDNA in the following reverse transcription system: 1 *μ*l enzyme mix, 1 *μ*l RT primer mix, 4 *μ*l 5x PrimeScript buffer, 2.7 *μ*g RNA, and 20 *μ*l RNase-free H_2_O. The reaction procedure included 37°C lasting 15 minutes, 85°C lasting 5 seconds, and 4°C which was held. RT-qPCR was carried out in accordance with the following reaction system: 1 *μ*l forward primer, 1 *μ*l reverse primer, 10 *μ*l 2x mix, 7 *μ*l H_2_O, and 1 *μ*l cDNA. Primer sequences were as follows: GAPDH, 5′-GCCGAGGAGGTCAACTACAT-3′ (forward), 5′-GCTCAGGGTGATTGCGTAT-3′ (reverse); ALOX15, 5′-CCCTGGATAAGGAAATTGAGATCC-3′ (forward), 5′-CCCTGGAGGAAATTGAGATCC-3′ (reverse); ENST00000538705.1, 5′-TTGCCTTTCTTGCAAAGTTTCC-3′ (forward), 5′-CACTTTCCCTTTTCTACTTGCTCG-3′ (reverse); ENST00000556936.1, 5′-CTTCCTTGTTCTGCTCTGGTTG-3′ (forward), 5′-ACCCTAATGAACGATGTCACCC-3′ (reverse); and U6, 5′-CTCGCTTCGGCAGCACA-3′ (forward), 5′-AACGCTTCACGAATTTGCGT-3′ (reverse). Relative expression was determined with 2^-*ΔΔ*Ct^ method.

### 2.4. Cell Culture and Transfection

Human coronary artery endothelial cells (HCAECs) were purchased from Jiangsu Punuosheng Biotechnology Co., Ltd. (China). HCAECs were maintained in endothelial basal medium 2 (EBM-2) supplemented with 15% fetal bovine serum (FBS; HyClone, USA). All HCAECs were cultured in an incubator with a humid environment and 5% CO_2_ at 37°C. siRNA sequences were as follows: siRNA negative control (si-NC): 5′-UUCUCCGAACGUGUCACGUTT-3′ (forward), 5′-ACGUGACACGUUCGGAGAATT-3′ (reverse); si-ALOX15#1: 5′-CGCUAUCAAAGACUCUCUAAATT-3′ (forward), 5′-UUUAGAGAGUCUUUGAUAGCGTT-3′ (reverse); si-ALOX15#2: 5′-AUGACUUCAACCGGAUUUUCUTT-3′ (forward), 5′-AGAAAAUCCGGUUGAAGUCAUTT-3′ (reverse); si-ALOX15#3: 5′-GUCGAUACAUCCUAUCUUCAATT-3′ (forward), 5′-UUGAAGAUATGGAU (reverse); si-ENST00000538705.1#1: 5′-UGCUUGUUUUAUUAUGUUUUCUTT-3′ (forward), 5′-AGAAAACAUAAUAAACAAGCATT-3′ (reverse); si-ENST00000538705.1#2: 5′-CACUCAAUAAAUAUUUUG-3′ (forward), 5′-AGCAAAAAUAUUUAUUGAGUGTT-3′ (reverse); and si-ENST00000538705.1#3: 5′-CCCCAUUUUAAUCUUUCAGUATT-3′ (forward), 5′-UACUGAAAGAUUAAAAUGGGGTT-3′ (reverse). The above siRNAs were transfected into HCAECs with the Lipofectamine RNAiMAX reagent (Thermo Scientific, USA). After 48 h, RT-qPCR was presented for measuring the expression of ALOX15 and ENST00000538705.1.

### 2.5. Western Blotting

Protein extraction was presented, and protein concentration was measured with the BCA method. Thereafter, protein was separated with SDS-PAGE electrophoresis and transferred onto the PVDF membrane. The membrane was sealed by 5% milk/TBST at room temperature for 1 h, followed by incubation with primary antibodies against ALOX15 (1 : 1000; 10021-1-Ig; Proteintech, China) and GAPDH (1 : 1500; 60004-1-Ig; Proteintech) at 4°C overnight. Thereafter, the membrane was incubated by HRP-labeled goat antirabbit secondary antibody (1 : 2000; SA00001-2; Proteintech) at room temperature for 1 h. Protein bands were developed with the ECL reagent and quantified with ImageJ software.

### 2.6. Cell Counting Kit-8 (CCK-8)

HCAECs in the logarithmic phase were digested and resuspended in complete medium to a concentration of 3.5 × 10^4^/ml. Thereafter, HCAECs were inoculated into a 96-well plate (3500 cells/well) and incubated for 18 h for later use. HCAECs were transfected with specific siRNAs and cultured for 24 h, 36 h, 48 h, and 72 h. 10 *μ*l CCK-8 solution was added to each well as well as incubated at 37°C for 4 h. After adding 10 *μ*l of stop solution to each well, the optical density (OD) value at 450 was measured with a microplate reader.

### 2.7. Wound-Healing Assay

HCAECs were plated into a 6-well plate at 3∗10^5^ cells/well and incubated overnight in an incubator with 5% CO_2_ at 37°C. After siRNA transfection for 24 h, a 10 *μ*l pipette tip was utilized to make cell scratches perpendicular to the well plate. The cell culture fluid was aspirated, and the well plate was washed three times with PBS to wash away the cell debris generated by the scratch. Thereafter, the corresponding serum-free medium was added. Images were acquired at 0 h, 6 h, 24 h, and 48 h, respectively.

### 2.8. Animals

Twenty healthy adult male Sprague-Dawley rats (age 8-10 weeks; body weight 250-300 g) were purchased from the Hangzhou Scientific Cloud Biotechnology Co., Ltd. (China). According to the National Institutes of Health “Guidelines for the Care and Use of Laboratory Animals” (Bethesda, Maryland, USA), the rats were kept in the animal room at 25 ± 1°C. All rats had a standard diet and no dietary restrictions. Our study gained the approval of the Animal Ethics Committee of the Third Affiliated Hospital of Shanghai University (Wenzhou People's Hospital) (KY-2017029).

### 2.9. Construction of Myocardial Infarction (MI) Rat Models

All rats were randomly divided into the sham operation group, MI+si-NC group, MI+si-ENST00000538705.1 group, and MI+si-ALOX15 group (*n* = 5 each group). The rats were anesthetized with intraperitoneal injection of pentobarbital sodium (50 mg/kg). Thereafter, their limbs were fixed, the trachea was intubated, and the electrocardiograph and respirator were connected. A 1.5 cm incision was made on the left side of the chest between the third and fourth ribs for thoracotomy. The left atrial appendage was slightly lifted with ophthalmic curved forceps. The left anterior descending artery was ligated with 5/0 surgical sutures about 1 mm below the conus artery and then sutured to close the chest. The markedly elevated ST segment in ECG lead II indicated a successful ligation. For the MI+si-NC group, the rats received a single intramyocardial injection of lentiviral therapy with a blank sequence 30 minutes before surgery. For the MI+si-ENST00000538705.1 group, the rats were treated with intramyocardial injection of 50 *μ*g of lentivirus with the si-ENST00000538705.1 sequence 30 minutes before surgery. For the MI+si-ALOX15 group, the rats were treated with 50 *μ*g lentivirus with si-ALOX15 sequence intramyocardial injection 30 minutes before surgery. For the sham operation group, the rats received only unligated surgical thread insertion. After two weeks, the rats were euthanized by intraperitoneal injection of 200 mg/kg pentobarbital sodium, and the rat hearts were excised and fixed with 4% paraformaldehyde or stored at 80°C.

### 2.10. Histological Analysis

Myocardial tissues were fixed in 4% paraformaldehyde for more than 24 hours, then dehydrated and embedded in paraffin. Subsequently, the paraffin-embedded tissues were cut into 4 *μ*m. After dewaxing into water, the sections were stained with Harris hematoxylin (Proteintech, China) for 10 minutes. After rinsing with tap water, the slices were differentiated with 1% hydrochloric acid alcohol for a few seconds. After rinsing with tap water for 10 minutes, the sections were turned blue with PBS for 5 minutes. The sections were then stained in eosin (Sigma, USA) staining solution for 3 minutes. After dehydration and mounting, microscopic examination, image acquisition, and analysis were presented.

### 2.11. Biochemical Tests

Biochemical tests were presented strictly in accordance with the corresponding instructions of high-density lipoprotein (HDL; K076(2019005); Changchun Huili Biotechnology Co., Ltd., China), total cholesterol (TC; C063(2019006); Changchun Huili Biotechnology Co., Ltd., China), and low-density lipoprotein (LDL; K076(2019005); Changchun Huili Biotechnology Co., Ltd., China) kits. The antigen was diluted to 0.1 ml/well with a carbonate buffer and incubated overnight at 4°C. The serum samples were washed three times the next day, and then, the diluted supernatant (0.1 ml) was added to the reaction well. The samples were then incubated at 37°C for 1 hour. Blank, negative, and positive wells were set to compare with reaction wells. A freshly diluted enzyme-labeled secondary antibody was added and incubated at 37°C for 50 minutes. Next, the holes were washed with deionized distilled water. Temporarily prepared tetramethylbenzidine was added to each reaction well and incubated at 37°C for 20 minutes. Stop solution (50 *μ*l) was added to stop the reaction. The OD value was detected at 450 nm wavelength within 20 minutes.

### 2.12. Statistical Analysis

All statistical analyses were conducted by the R software and GraphPad Prism 8.0 software. Each experiment was repeated at least three times. The data are expressed as mean ± standard deviation. The differences between different groups were analyzed by Student's *t*-test or one-way analysis of variance (ANOVA). *p* < 0.05 was considered statistically significant.

## 3. Results

### 3.1. Identification ACS-Specific Circulating lncRNAs and mRNAs

**Figure 1:**
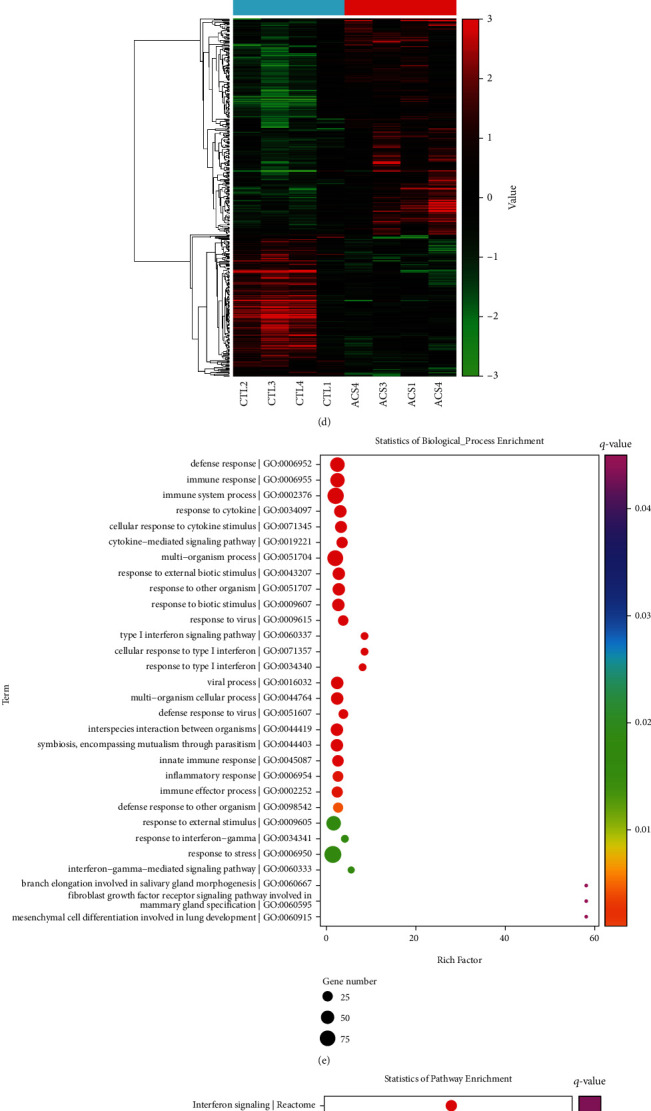
Identification of ACS-specific circulating lncRNAs and mRNAs through microarrays. (a, b) Volcano and heat map diagrams show dysregulated circulating lncRNAs in ACS patients compared with healthy subjects. (c, d) Volcano and heat map diagrams visualize dysregulated circulating mRNAs in ACS patients compared with healthy subjects. (e–g) The first 30 enrichment results of (e) biological processes, (f) KEGG pathways, and (g) diseases enriched by dysregulated circulating mRNAs. Rich factor indicates the ratio of input frequency/background frequency, the size of the bubble indicates the number of dysregulated circulating mRNAs, and the color corresponds to the *q*-value.

This study presented a microarray analysis between three pairs of serum samples from ACS patients and healthy subjects. With |log2fold − change| > 1 and *p* < 0.05, we determined 111 upregulated lncRNAs and 242 downregulated lncRNAs in ACS patients compared with healthy controls (Figures 1(a) and 1(b)). Meanwhile, 266 mRNAs were upregulated, and 175 mRNAs were downregulated in ACS (Figures 1(c) and 1(d)). In Figure 1(e), we found that immune-related biological processes (immune system process, response to cytokine, cellular response to cytokine stimulus, and cytokine-mediated signaling pathway, etc.) were prominently enriched by dysregulated mRNAs. KEGG enrichment analysis demonstrated that these dysregulated mRNAs were linked to immune activation pathways such as cytokine signaling in the immune system, cytokine-cytokine receptor interaction, and interleukin signaling pathway (Figure 1(f)). Also, the above mRNAs were related to diverse diseases (Figure 1(g)).

### 3.2. Analysis of Up- and Downstream Factors of Dysregulated lncRNAs in ACS

**Figure 2:**
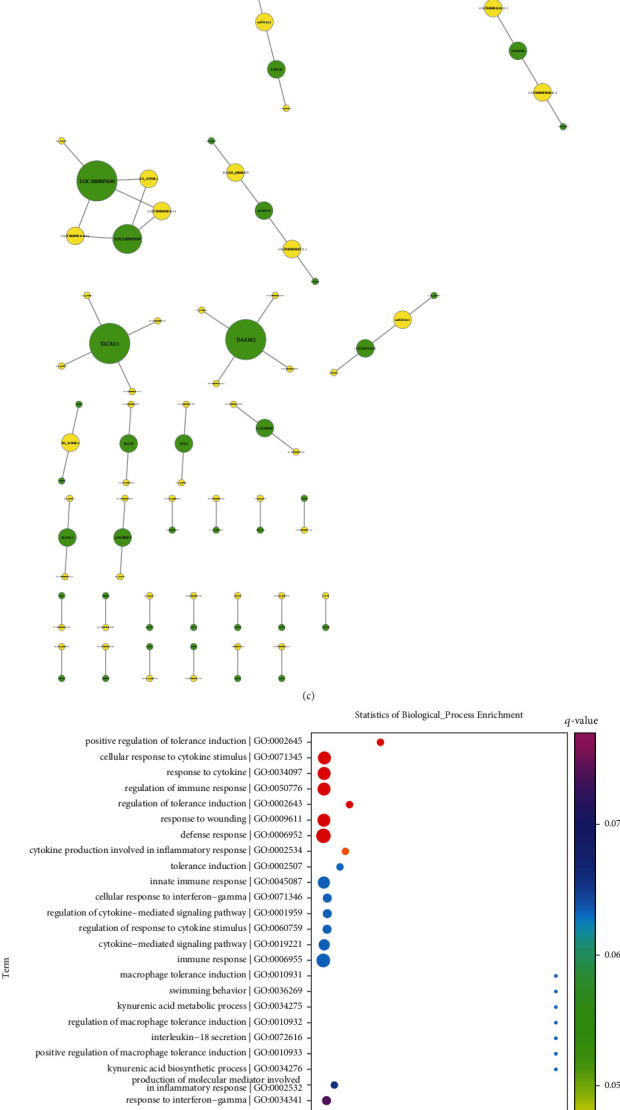
Analysis of up- and downstream factors of dysregulated lncRNAs in ACS. (a) The network of transcription factors and dysregulated lncRNAs. The yellow circle represents lncRNA, the purple circle represents transcription factor, and the size of the circle represents the degree of transcription factor or lncRNA in the network. (b) The lncRNA-mRNA coexpression network. The yellow circle represents lncRNA, the green circle represents mRNA, and the size of the circle represents the degree of lncRNA or mRNA in the network. The red line represents a positive correlation, and the blue line represents a negative correlation. (c) The network of lncRNAs and their downstream targets. The yellow circle indicates lncRNA, the green circle indicates downstream mRNA, and the size of the circle represents the degree of lncRNA or downstream target in the network. (d–f) The first 30 enrichment results of (d) biological processes, (e) KEGG pathways, and (f) diseases of downstream targets enriched by dysregulated lncRNAs. The rich factor indicates the ratio of input frequency/background frequency, the size of the bubble indicates the number of genes annotated to this function entry for downstream genes, and the color corresponds to the *q*-value.

Transcription factor prediction was presented utilizing the Match-1.0 Public transcription factor prediction tool. The binding of the 2000 bp upstream and 500 bp downstream region of the start site of each lncRNA to the transcription factor was predicted, and the results are shown in Figure 2(a). Coexpression analysis adopted the standardized signal value of each probe in each sample as the data source to perform pairwise correlation calculation and hypothesis verification, thereby obtaining the correlation coefficient and *p* value. In this study, lncRNA-mRNA coexpression pairs were determined in accordance with |correlation coefficient| > 0.99 and *p* value < 0.05, as shown in Figure 2(b). Additionally, we carried out the coexpression analysis of lncRNA and mRNA. Briefly, cis-prediction was used for exploring lncRNA-mRNA pairs within 10 kb of the genome while trans-prediction adopted the blat tool to compare the lncRNA and mRNA (3′UTR) sequences to screen lncRNA-mRNA pairs with similar sequences.  Figure 2(c) depicted the interaction network of lncRNAs and their coexpressed mRNAs with |correlation coefficient| > 0.99 and *p* value < 0.05. Biological significance of coexpressed mRNAs was further probed. In Figure 2(d), the above mRNAs were remarkably enriched in immune-relevant biological processes such as regulation of immune response, cellular response to cytokine stimulus, cytokine production involved in inflammatory response, innate immune response, cellular response to interferon-gamma, regulation of cytokine-mediated signaling pathway, macrophage tolerance induction, IL-18 secretion, and regulation of IL-12 production. Additionally, immune-relevant pathways were prominently enriched by coexpressed mRNAs, including cytokine signaling in the immune system, regulation of IFN*α* signaling, and IL-6 signaling (Figure 2(e)). Also, we found that these mRNAs were distinctly linked to diverse diseases (Figure 2(f)).

### 3.3. Verification of the Expression of ENST00000538705.1, ENST00000556936.1, and ALOX15 in ACS Patients

**Figure 3:**
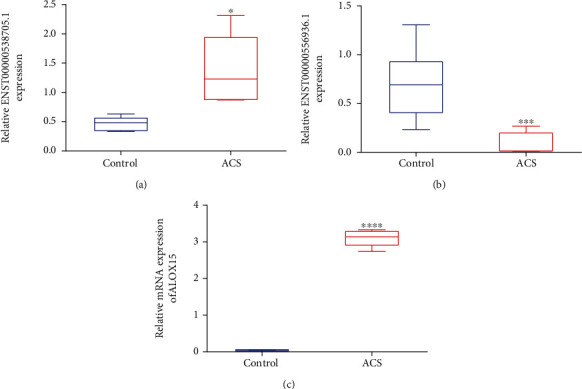
Verification of the expression of ENST00000538705.1, ENST00000556936.1, and ALOX15 in ACS patients. (a–c) RT-qPCR for measuring the expression of (a) ENST00000538705.1, (b) ENST00000556936.1, and (c) ALOX15 in serum samples of ACS patients and healthy subjects. ^∗^*p* < 0.05, ^∗∗∗^*p* < 0.001, and ^∗∗∗∗^*p* < 0.0001.

The expression of lncRNAs ENST00000538705.1 and ENST00000556936.1 as well as coexpressed mRNA ALOX15 was further verified in serum specimens of ACS patients and healthy subjects. Our data confirmed that the lncRNA ENST00000538705.1 expression was remarkably upregulated as well as the ENST00000556936.1 expression was prominently downregulated in ACS patients compared with healthy subjects (Figures 3(a) and 3(b)). Additionally, we found the prominent upregulation of ALOX15 in ACS patients (Figure 3(c)).

### 3.4. Silencing ENST00000538705.1 Reduces ALOX15 Expression in HCAECs

**Figure 4:**
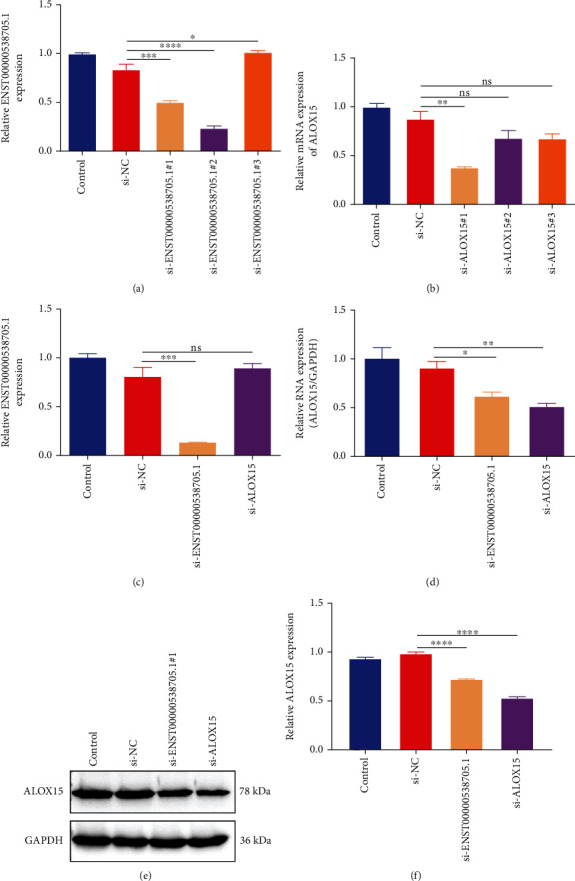
Silencing ENST00000538705.1 reduces ALOX15 expression in HCAECs. (a) RT-qPCR for evaluating the transfection effect of three specific siRNAs against ENST00000538705.1 in HCAECs. (b) The transfected effect of three specific siRNAs against ALOX15 in HCAECs via RT-qPCR. (c) RT-qPCR for measuring the expression of ENST00000538705.1 in HCAECs transfected with si-ENST00000538705.1 or si-ALOX15. (d–f) RT-qPCR and western blotting for quantifying the expression of ALOX15 in HCAECs transfected with si-ENST00000538705.1 or si-ALOX15. ns: not significant; ^∗^*p* < 0.05, ^∗∗^*p* < 0.01, ^∗∗∗^*p* < 0.001, and ^∗∗∗∗^*p* < 0.0001.

To investigate the interaction of ENST00000538705.1 and ALOX15 as well as their biological significance, HCAECs were separately transfected with specific siRNAs against ENST00000538705.1 and ALOX15. In Figure 4(a), si-ENST00000538705.1#2 remarkably lowered the expression of ENST00000538705.1 in HCAECs, which was adopted for further experiments. Meanwhile, we investigated the excellent knockdown effect of si-ALOX15#1 in HCAECs (Figure 4(b)). Further analysis indicated that silencing ALOX15 did not alter the ENST00000538705.1 expression in HCAECs (Figure 4(c)). However, ENST00000538705.1 knockdown remarkably reduced the expression of ALOX15 in HCAECs (Figures 4(d)–4(f)). This indicated that ALOX15 acted as a downstream target of ENST00000538705.1.

### 3.5. Silencing ENST00000538705.1 or ALOX15 Weakens the Proliferative and Migrated Capacities of HCAECs

**Figure 5:**
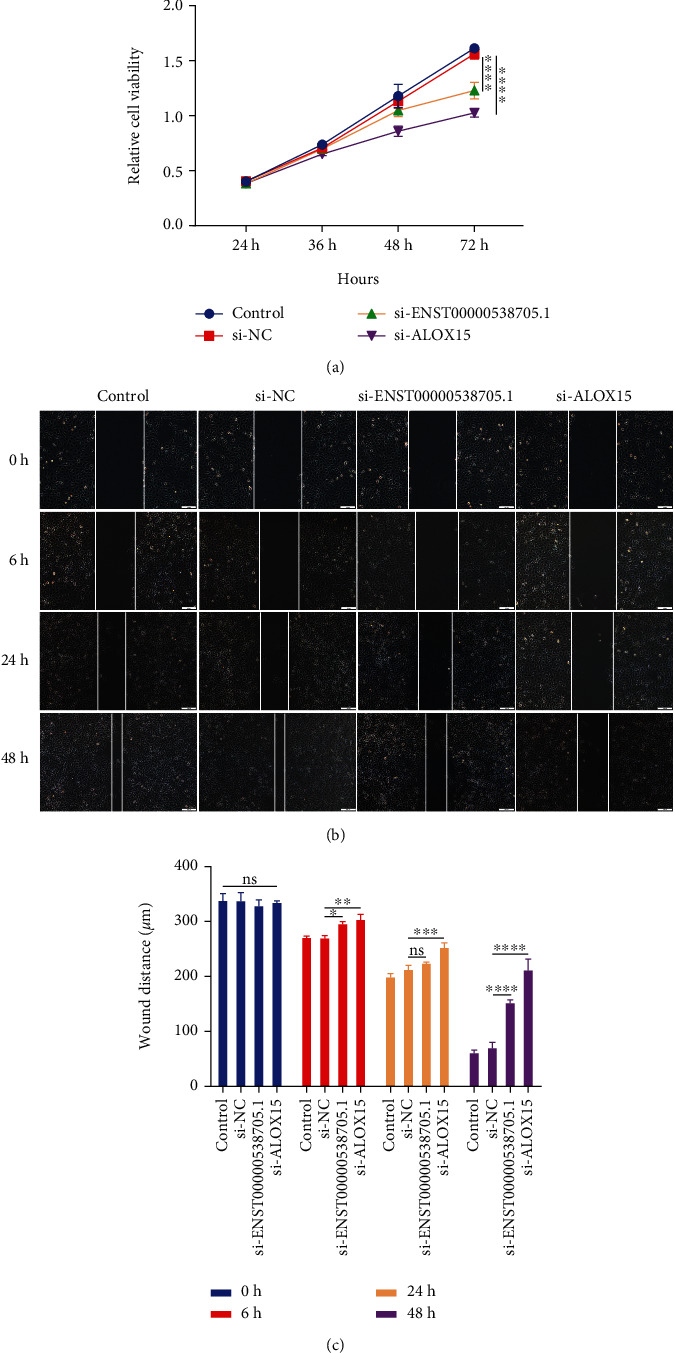
Silencing ENST00000538705.1 or ALOX15 weakens the proliferative and migrated capacities of HCAECs. (a) CCK-8 for measuring viable HCAECs transfected with si-ENST00000538705.1 or si-ALOX15 in specified time points. (b, c) Migration capacities of HCAECs transfected with si-ENST00000538705.1 or si-ALOX15 through a wound-healing assay in specified time points. Bar = 100 *μ*m. Magnification = 200x. ns: not significant; ^∗^*p* < 0.05, ^∗∗^*p* < 0.01, ^∗∗∗^*p* < 0.001, and ^∗∗∗∗^*p* < 0.0001.

CCK-8 results demonstrated that the viable HCAECs transfected with si-ENST00000538705.1 or si-ALOX15 were markedly decreased in comparison to the si-NC group (Figure 5(a)). This indicated that silencing ENST00000538705.1 or ALOX15 remarkably weakened the proliferation of HCAECs. A wound-healing assay was presented to evaluate the migration of HCAECs. Compared with the si-NC group, the migration ability of HCAECs transfected with si-ENST00000538705.1 or si-ALOX15 was remarkably weakened (Figures 5(b) and 5(c)).

### 3.6. Silencing ENST00000538705.1 or ALOX15 Improves Myocardial Injury in Rats with MI

**Figure 6:**
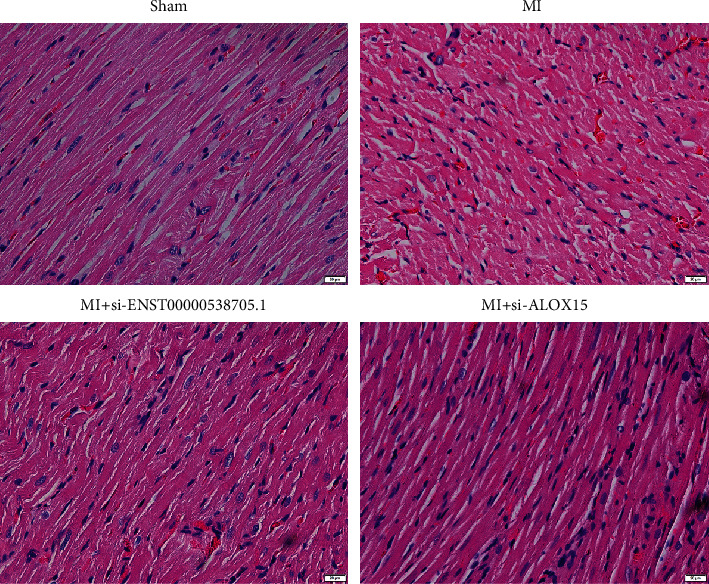
Silencing ENST00000538705.1 or ALOX15 improves myocardial injury of MI rats. H&E staining for observing the pathological condition of myocardial tissues of rats in the sham group, MI group, MI+si-ENST00000538705.1 group, and MI+ALOX15 group. Bar = 20 *μ*m. Magnification = 200x.

To further investigate the effects of ENST00000538705.1 and ALOX15 on ACS, we constructed MI rat models through ligating the left coronary artery. Two weeks later, the heart tissues were taken for H&E to observe the pathological condition. The cardiomyocytes of rats in the sham operation group were arranged more uniformly, without breaks and with normal cardiomyocytes (Figure 6). However, the cardiomyocytes of the MI model group were swollen and obviously thickened, with very irregular shapes and disordered arrangement. For MI rats with si-ENST00000538705.1 or si-ALOX15 treatment, cardiomyocyte hypertrophy and arrangement disorder were remarkably ameliorated.

### 3.7. Silencing ENST00000538705.1 or ALOX15 Reduces Blood Lipids in Rats with MI

**Figure 7:**
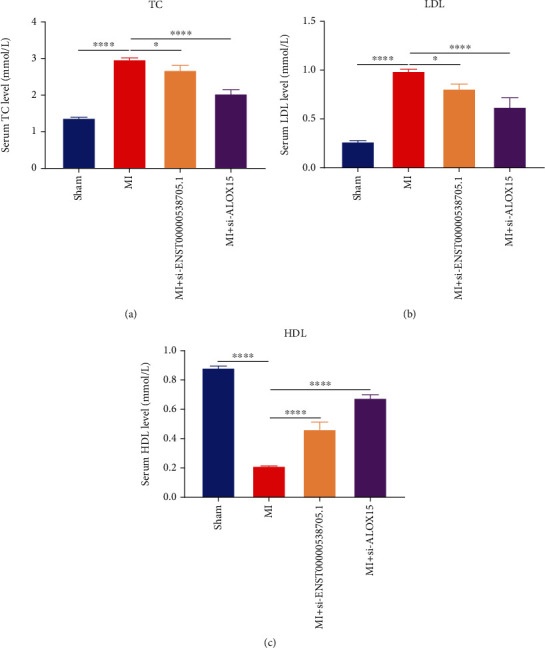
Silencing ENST00000538705.1 or ALOX15 reduces blood lipids of MI rats. (a–c) The levels of (a) TC, (b) LDL, and (c) HDL in the serum of rats in the sham group, MI group, MI+si-ENST00000538705.1 group, and MI+ALOX15 group. ^∗^*p* < 0.05 and ^∗∗∗∗^*p* < 0.0001.

The serum levels of TC, LDL, and HDL of rats in each group were detected separately. Compared with rats in the sham operation group, the serum levels of TC and LDL in MI rats were significantly increased (Figures 7(a) and 7(b)). Compared with rats with MI, after intervention with si-ENST00000538705.1 or si-ALOX15, the serum levels of TC and LDL of MI rats were significantly decreased. As shown in Figure 7(c), in comparison to rats in the sham operation group, the serum levels of HDL of MI rats were significantly reduced. However, the serum HDL levels of MI rats were significantly increased following treatment with si-ENST00000538705.1 or si-ALOX15.

### 3.8. Silencing ENST00000538705.1 or ALOX15 Reduced their Expression Levels in the Serum of Rats with MI

**Figure 8:**
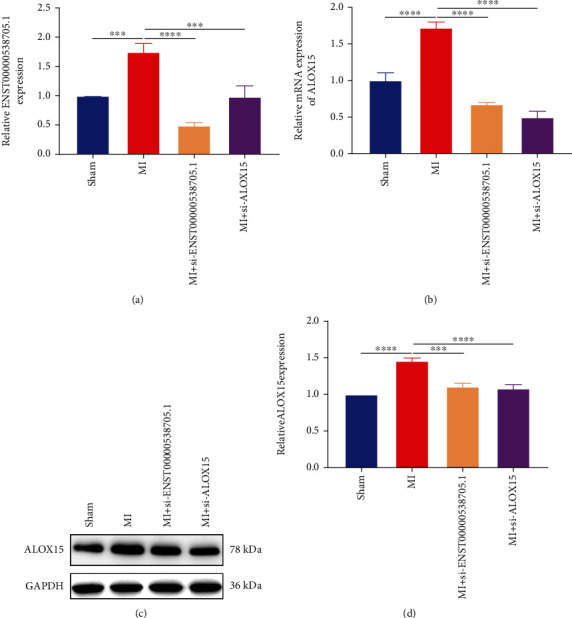
Silencing ENST00000538705.1 or ALOX15 reduces their expression levels in the serum of rats with MI. (a) ENST00000538705.1 expression in the serum of rats in the sham group, MI group, MI+si-ENST00000538705.1 group, and MI+ALOX15 group through RT-qPCR. (b–d) RT-qPCR and western blotting for measuring the expression of ALOX15 in the serum specimens from above groups. ^∗∗∗^*p* < 0.001 and ^∗∗∗∗^*p* < 0.0001.

The expression level of ENST00000538705.1 was further measured. The results showed that ENST00000538705.1 presented a higher expression in the serum of rats in the MI group than in the sham operation group. Nevertheless, its expression in the serum of MI rats was remarkably lowered after administration with si-ENST00000538705.1 or si-ALOX15 (Figure 8(a)). Also, ALOX15 expression was detected in serum samples. As a result, higher ALOX15 expression was investigated in the MI group in comparison to the sham group (Figures 8(b)–8(d)). But intervention with si-ENST00000538705.1 or si-ALOX15 decreased the serum levels of ALOX15 for MI rats.

## 4. Discussion

In the past few decades, the molecular mechanisms of ACS have been extensively studied [21–24]. Nevertheless, in-depth analysis is needed to uncover the pathogenesis of ACS. Although a few thousands of lncRNAs have been functionally characterized in ACS, their potential mechanisms in ACS remain greatly indistinct [25–27]. In our study, we adopted microarray technology to obtain the expression data of lncRNAs and mRNAs in serum specimens from 4 patients with ACS and 4 healthy controls. As a result, we determined 111 upregulated lncRNAs and 242 downregulated lncRNAs in ACS. Additionally, 266 upregulated mRNAs and 175 downregulated mRNAs were determined in ACS. Immune and inflammation dysfunctions exert crucial roles in clinical manifestations and complications of ACS [3]. Our functional enrichment results showed that dysregulated circulating mRNAs were remarkably linked to immune-relevant biological processes and pathways, indicating their roles in ACS progression.

The regulatory mechanisms of these dysregulated lncRNAs were further probed out. We firstly analyzed the upstream regulatory mechanism of dysregulated lncRNAs. We identified the transcription factors of these lncRNAs, such as Nkx2-5, Pax-4, HNF-4, FOXJ2, and CHOP-C/EBPalpha. These transcription factors might be involved in modulating the expression of circulating lncRNAs in ACS. Coexpression analysis is based on correlation, looking for lncRNA-mRNA relationship pairs with similar expression profiles from the genetic expression layer [28]. Many functionally related genes have very similar expression profiles under a set of related conditions, especially genes that are coregulated by common transcription factors, or their products form the same protein complex or participate in the same regulatory pathway [29]. In this study, our coexpression analysis and network construction based on coexpression results can help us discover the possible relationship between lncRNA and mRNA, determine the lncRNA that affects the regulation of the mRNA expression, and find the lncRNA that plays a central regulatory role in the network and discovers the possible new mechanism of action of lncRNA. It has been demonstrated that lncRNA can regulate the expression of nearby genes through interaction with nearby genes or regulate the expression of distant genes through the indirect influence of miRNA [30–32]. Shen et al. identified key molecular markers (PDZK1IP1, PROK2, LAMP3, etc.) of ACS utilizing peripheral blood transcriptome sequencing and mRNA-lncRNA coexpression analysis [33]. Herein, we presented target gene prediction analysis on the basis of the above two mechanisms of action to predict the mRNA that lncRNA may modulate. Target gene prediction can be divided into cis-prediction and trans-prediction. For cis-prediction, we predicted the lncRNA-mRNA relationship pairs that had a certain relationship through the position alignment of lncRNA and mRNA. Meanwhile, for trans-prediction, possible lncRNA-mRNA relationship pairs were determined through sequence alignment. Overall, our analysis characterized the up- and downstream regulatory mechanisms of dysregulated lncRNAs.

Among lncRNA-mRNA relationship pairs, we focused on ENST00000538705.1 and ALOX15. After verification, circulating ENST00000538705.1 and ALOX15 were both upregulated in ACS patients. In HCAECs, we found that silencing ENST00000538705.1 remarkably decreased the expression ALOX15. Nevertheless, ALOX15 knockdown did not influence the ENST00000538705.1 expression in HCAECs. This indicated that ALOX15 might be a downstream target of ENST00000538705.1. The proliferation and migration of endothelial cells following MI are crucial for angiogenesis [34]. Previously, Liu et al. demonstrated that lncRNA ANRIL suppression enabled to promote cell proliferation and tubule formation and inhibit inflammatory activation and apoptosis of endothelial cells [35]. Du et al. reported that lncRNA HCG11 alleviated high glucose-induced vascular endothelial damage by increasing cell proliferation and tube formation [26]. Additionally, lncRNA TCONS_00024652 was identified to facilitate vascular endothelial cell proliferation and angiogenesis [36]. Herein, we found that silencing ENST00000538705.1 and ALOX15 markedly weakened the proliferation and migration of HCAECs, indicating that they might participate in ACS progression. Evidence has demonstrated that the effect of ALOX15 induces endothelial cell barrier dysfunction in high-fat-diet rats [37]. Hence, we speculated that the antisense lncRNA ENST00000538705.1 might enhance the stability of ALOX15 mRNA, thereby enhancing the proliferation and migration ability of HCAECs.

The therapeutic effects of si-ENST00000538705.1 and si-ALOX15 were also investigated in MI rat models. Our results demonstrated that silencing ENST00000538705.1 or ALOX15 remarkably relieved myocardial injury following MI. Previously, lncRNA SLC8A1-AS1 protected the myocardium from damage via weakening SLC8A1 and activating cGMP-PKG signaling in MI models [38]. Moreover, ATP2B1-AS1 knockdown protected against MI mice through blocking NF-*κ*B signaling [27]. Our study noted that ENST00000538705.1 or ALOX15 knockdown reduced serum TC and LDL levels as well as elevated serum HDL levels in MI rats. These results indicated that ENST00000538705.1 and ALOX15 might become potential targets for the treatment of MI.

The limitations of our study should be pointed out. First of all, the sample size used for microarray and RT-qPCR analysis was relatively small. In our future studies, the sample size of each group will be increased for further verifying our findings. Furthermore, the molecular mechanisms underlying ENST00000538705.1 and ALOX15 in ACS progression remain to be fully elucidated, and in-depth studies are required.

## 5. Conclusion

In conclusion, we determined upregulated ENST00000538705.1 and ALOX15 in serum specimens of patients with ACS. Bioinformatics analysis identified the remarkable interaction between ENST00000538705.1 and ALOX15, and ENST00000538705.1 knockdown significantly inhibited ALOX15 expression in HCAECs. Silencing ENST00000538705.1 and ALOX15 both weakened the proliferation and migration of HCAECs. Additionally, their knockdown relieved myocardial damage of MI rats. Altogether, lncRNA ENST00000538705.1 and ALOX15 may become potential molecular targets for ACS therapy.

## Data Availability

The datasets analyzed during the current study are available from the corresponding authors on reasonable request.

## Abbreviations

ACS: Acute coronary syndrome
AMI: Acute myocardial infarction
MI: Myocardial infarction
ncRNA: Noncoding RNA
lncRNA: Long ncRNA
RT-qPCR: Real-time quantitative PCR
HCAECs: Human coronary artery endothelial cells
FBS: Fetal bovine serum
NC: Negative control
CCK-8: Cell counting kit-8
OD: Optical density
HDL: High-density lipoproteins
TC: Total cholesterol
LDL: Low-density lipoproteins
One-way ANOVA: One-way analysis of variance.
